# Identifying Optimal Vaccination Strategies for Serogroup A *Neisseria meningitidis* Conjugate Vaccine in the African Meningitis Belt

**DOI:** 10.1371/journal.pone.0063605

**Published:** 2013-05-09

**Authors:** Sara Tartof, Amanda Cohn, Félix Tarbangdo, Mamoudou H. Djingarey, Nancy Messonnier, Thomas A. Clark, Jean Ludovic Kambou, Ryan Novak, Fabien V. K. Diomandé, Isaïe Medah, Michael L. Jackson

**Affiliations:** 1 Meningitis and Vaccine Preventable Diseases Branch, Centers for Disease Control and Prevention, Atlanta, Georgia, United States of America; 2 Direction de la Lutte Contre la Maladie, Ministère de la Santé, Ouagadougou, Burkina Faso; 3 Intercountry Support Team for West Africa, World Health Organization, Ouagadougou, Burkina Faso; 4 WHO Intercountry Support Team for West Africa, Ouagadougou, Burkina Faso; 5 Group Health Research Institute, Group Health Cooperative, Seattle, Washington, United States of America; Health Protection Agency, United Kingdom

## Abstract

**Objective:**

The optimal long-term vaccination strategies to provide population-level protection against serogroup A Neisseria meningitidis (MenA) are unknown. We developed an age-structured mathematical model of MenA transmission, colonization, and disease in the African meningitis belt, and used this model to explore the impact of various vaccination strategies.

**Methods:**

The model stratifies the simulated population into groups based on age, infection status, and MenA antibody levels. We defined the model parameters (such as birth and death rates, age-specific incidence rates, and age-specific duration of protection) using published data and maximum likelihood estimation. We assessed the validity of the model by comparing simulated incidence of invasive MenA and prevalence of MenA carriage to observed incidence and carriage data.

**Results:**

The model fit well to observed age- and season-specific prevalence of carriage (mean pseudo-R2 0.84) and incidence of invasive disease (mean R2 0.89). The model is able to reproduce the observed dynamics of MenA epidemics in the African meningitis belt, including seasonal increases in incidence, with large epidemics occurring every eight to twelve years. Following a mass vaccination campaign of all persons 1–29 years of age, the most effective modeled vaccination strategy is to conduct mass vaccination campaigns every 5 years for children 1–5 years of age. Less frequent campaigns covering broader age groups would also be effective, although somewhat less so. Introducing conjugate MenA vaccine into the EPI vaccination schedule at 9 months of age results in higher predicted incidence than periodic mass campaigns.

**Discussion:**

We have developed the first mathematical model of MenA in Africa to incorporate age structures and progressively waning protection over time. Our model accurately reproduces key features of MenA epidemiology in the African meningitis belt. This model can help policy makers consider vaccine program effectiveness when determining the feasibility and benefits of MenA vaccination strategies.

## Introduction

The greatest burden of meningococcal disease worldwide occurs in an area of sub-Saharan Africa known as the meningitis belt, which stretches from Senegal in the west to Ethiopia in the east [1]. Major epidemics of meningococcal meningitis have occurred in this region for more than 100 years, where the epidemiology is characterized by localized annual minor epidemics as well as major epidemic waves every eight to twelve years [2], [3]. Epidemics occur during dry seasons which run from December through May and incidence declines predictably with the onset of rains in the rainy season [4]. Most cases occur in persons between 1 and 29 years of age and approximately 90% of cases during epidemics are caused by serogroup A *Neisseria meningitidis* (MenA) [5]–[8]. Bacteria are transmitted from person-to-person through droplets of respiratory or throat secretions, primarily from asymptomatic carriers. Invasive disease is a rare outcome, while carriage prevalence estimates of *N. meningitidis* in the meningitis belt range from 3% to 30%, varying by age and season [9], [10].

A new meningococcal serogroup A polysaccharide-tetanus toxoid conjugate vaccine (PsA-TT, MenAfriVac™) was developed to eliminate epidemic meningitis in sub-Saharan Africa [11], [12]. The vaccine is priced at 40 cents a dose to be accessible to countries in the meningitis belt. Pre-licensure clinical trials demonstrated high immunogenicity after a single dose among persons aged 1 through 29 years [13], [14]. This conjugate vaccine has potential to reduce nasopharyngeal carriage and induce herd immunity in the population [15]. Burkina Faso is a landlocked country of approximately 16 million people that is located entirely in the meningitis belt. In addition to the characteristic epidemics of the region, Burkina Faso experiences hyper-endemic rates of meningitis [16] [17]. In December of 2010, Burkina Faso became the first country to implement mass vaccination of all 1 to 29 year olds with conjugate MenA vaccine. The campaign vaccinated over 11 million persons in ten days, with an administrative coverage of over 99% of the target population [18]. Early vaccine impact data of the 2011 season (the first year following the mass campaign) show significant declines in risk of meningitis and fatal meningitis in both vaccine eligible and ineligible age groups [19]. In 2011 and through week ten of 2012, zero cases of MenA meningitis occurred in vaccinated persons in Burkina Faso [20], [21], and no MenA carriage was detected among vaccinated individuals [15], [19].

Initial campaigns in countries introducing vaccine will target 1 to 29 year olds with a single dose of MenA conjugate vaccine. To achieve the greatest benefits from the new vaccine, it is critical to consider options for long-term vaccination strategies. In the years following an initial mass vaccination campaign, population-level susceptibility will return as immunity wanes and as herd immunity is diluted by new birth cohorts. Mathematical models are a useful and widely applied tool to illustrate and compare how different vaccine strategies are expected to perform in a modeled population [22], [23]. Therefore, we developed a model of MenA transmission and disease to explore the impact of different immunization strategies on MenA disease in Burkina Faso and the meningitis belt.

## Methods

### Model Structure and Population

**Figure 1:**
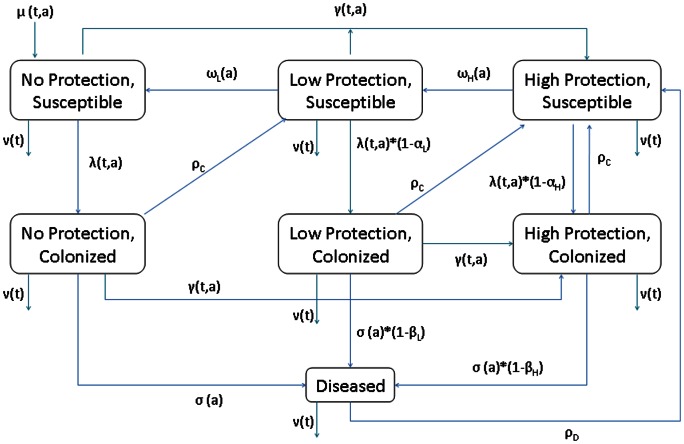
Model structure. Individuals are born into the no protection, susceptible state with time- and age- dependent birth rate *μ(t,a)* and die from all model states with time-dependent death rate *ν(t)*. Susceptible individuals become colonized at time- and age- dependent force of infection *λ(t,a)*, which is reduced by immunity due to low (*α_L_*) or high (*α_H_*) protection levels. Colonized individuals develop invasive disease at age-dependent rate *σ(a)*, which is reduced by low (*β_L_*) or high (*β_H_*) protective immunity. Diseased individuals recover to the high protection, susceptible state at recovery rate *ρ_D_*, low protection colonized individuals recover from colonization to the high protection, susceptible state at recovery rate *ρ_C,_* and no protection colonized individuals recover from colonization to the low protection, susceptible state at recovery rate *ρ_C_*. Protection wanes from high to low and from low to none, at age-dependent rate *ω_H_(a)* and *ω_L_*(a), respectively. Susceptible individuals with no or low protection are vaccinated at time- and age- dependent rate *γ(t,a)*, where vaccination induces high protection.

We adapted an existing model of *Haemophilus influenzae* type b (Hib) transmission to describe MenA transmission, colonization, and disease in Burkina Faso [22]. Briefly, we developed an age-structured mathematical model that divides the population into mutually exclusive states based on age, level of protection against MenA colonization and disease (High, Low, and None) and meningitis infection status (Susceptible, Colonized, and Diseased) (Figure 1). Like Hib, MenA transmission is primarily driven by asymptomatic carriers. For such pathogens, accurate modeling requires a model that can distinguish immunity to future invasive disease from immunity to future re-colonization (e.g. [22],[24],[25]). As such, the “high protection” states represents individuals who are have a relatively high level of immunity to becoming colonized and, if colonized, a high level of immunity to becoming diseased. The “low protection” states represent individuals with relatively low immunity to colonization, but still with fairly high immunity to disease if they become colonized. The “no protection” states represent individuals who have no immunity to colonization.

As our work with Hib showed, this model structure is well suited to pathogens such as MenA, where infectious asymptomatic carriers represent the majority of infections and where immunity to colonization wanes over time. This model can be expressed as a set of partial differential equations (Table S1), with rate parameters that dictate the movement of the population between the model states described above.

### Parameterization

Where possible, we set values for our model parameters using published and unpublished data (Table 1). We estimated birth and death rates and the age distribution of Burkina Faso based on Burkina Faso census data [26]. We estimated the rates of recovery from MenA colonization and disease from literature on the duration of MenA colonization [27], [28] and disease [7].

**Table 1:** Values and sources for parameters used in the model.

Parameter name	Value		Source(s)
**Rate of recovery from colonization**	12.175/year		[27]
**Rate of recovery from disease**	52.178/year		[7]
	***High***	***MenA Est.***	
**Immunity of high protection against colonization**	0.75	0.9	[30]
**Immunity of low protection against colonization**	0.25	0.5	No data available, best guess
**Immunity of high protection against disease (MenC)**	1.00		[33], [35]
**Immunity of low protection against disease (MenC)**	0.90		[29]
**Rate of waning from high to low protection:**	***MenC***	***MenA Est.***	
** <6 months of age**	0.877/year	0.439/year	Imputed from ratio of high/low to low/none waning in 6mo-2 yr
** 6 months–2 years of age**	0.285/year	0.143/year	[33]
** 3–10 years of age**	0.04/year	0.02/year	[34]
** ≥11 years of age**	0.025/year	0.013/year	[34]
**Rate of waning from low to no protection**	***MenC***	***MenA Est.***	
** <6 months of age**	0.4/year	0.2/year	[29]
** 6 months–2 years of age**	0.13/year	0.07/year	[29]
** 3–10 years of age**	0.04/year	0.02/year	[29]
** ≥11 years of age**	0.04/year	0.02/year	[29]
**Force of infection from outside population**	0.0005/year		[22]
**Birth rate per 1,000 population**	43.98/year		http://www.census.gov/population/international/data/idb/country.php
**Death rate per 1,000 population**	13.02/year		http://www.census.gov/population/international/data/idb/country.php
Rate of disease among colonized x+y*age			
** x Dry season**	0.0019/year		MLE
** y Dry season**	−0.0000104/year		MLE
** x Rainy season**	0.0018/year		MLE
** y Rainy season**	−0.0000110/year		MLE
**Rate of MenA vaccination**	89%		[9]
**Rate of EPI vaccination**	80%		[38], [39]

Data are limited on rates of waning protection against colonization, and against disease for colonized persons, after MenA infection or vaccination. We estimated protection against colonization and disease using antibody data from *N. meningitidis* serogroup C (MenC) conjugate vaccine studies, including studies which measured antibody titers post-vaccination and from vaccine effectiveness studies against carriage and disease [29], [30]. For our analyses, we used serum bactericidal antibody titers using rabbit complement (rSBA)> = 8 as the cut-off level for protection [31], [32]. We used primary series vaccination data with MenC conjugate vaccine to estimate immunity of the low protection state against disease, and data from booster vaccine doses to estimate immunity of the high protection state against colonization and disease. Data to estimate protection of low antibody against colonization were not available in the literature and were inferred to be one-third of protection against invasive disease for this study. Rates of protection waning were estimated from antibody titers or vaccine effectiveness estimates of MenC vaccine post-vaccination [29], [33]–[35]. The rate of waning was assumed to be constant from both high and low protection states. We estimated rates of waning from low to no protection based on vaccine effectiveness data following MenC primary series [29]. Rates of waning from high to low protection were estimated from studies reporting antibody titers following booster doses with MenC vaccine [33], [35]. Data were not available for the rate of waning from high to low protection in those aged less than six months of age and were imputed as being proportional to the ratio of high/low to low/none waning in the six month to two year old age group.

To estimate the age-specific rate at which disease develops in colonized persons, we used data on the incidence of MenA disease by age [19] [6] and the duration of disease to estimate the point prevalence of meningitis disease due to MenA. As a preliminary estimate of the age-specific rate of disease among colonized persons, we computed the age-specific ratio of colonization prevalence to MenA disease prevalence. We fit a variety of functions to this ratio and chose the function with the best fit to the data based on the Akaike Information Criteria. The preliminary estimate was then refined in the model fitting process, described below.

Currently, no published estimates are available of the force of infection (λ(t,a)), which is the rate at which susceptible individuals of age *a* become infected. To create estimates of the force of infection, we first partitioned the population into four age classes: less than 5 years; 5 to 12 years, 13 to 19 years, and 20 or more years of age. The force of infection on susceptibles in age class i at time *t* is then:
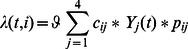
where j represents the four age classes; φ is a stochastic term; c_ij_ is the rate at which susceptibles in age class i contact persons in age class j; Y_j_ is the proportion of persons in age class j who are infectious; and p_ij_ is the probability of transmission from j to i given contact between susceptible and infectious persons [36]. The terms c_ij_ and p_ij_ can be combined into a single transmission coefficient, β_ij_. The collection of β_ij_ values forms a Who Acquires Infection from Whom (WAIFW) matrix. The stochastic term φ is added (from a random uniform distribution of 0 to 0.75) to allow cyclic but irregular epidemics similar to observed epidemic patterns. To reproduce the annual seasonality of carriage and disease, the force of infection on susceptibles must change seasonally. We operationalized this seasonality by having two different WAIFW matrices – one for the dry season, and one for the rainy season.

### Model Fitting

For this study, model fitting is the process of obtaining final values for the WAIFW matrices and the rate of disease among colonized persons. The goal of model fitting is to find parameter values that allow the model to reproduce the observed epidemiology of MenA in the meningitis belt. The observed data to which the model can be fit are:

- Age-specific prevalence of carriage during rainy seasons and a dry season with a minor epidemic;
- Age-specific incidence of invasive MenA disease during dry seasons with minor epidemics;
- Age-specific incidence of invasive MenA disease during dry seasons with major epidemics;
- Annual incidence rates of invasive MenA disease over time.

Incidence data was collected from national surveillance reports from Burkina Faso from 1998 to 2010. From 2004 to 2010, national surveillance data included counts of reported meningitis cases as well as laboratory testing results. However, prior to 2004, the surveillance system did not collect laboratory report data. Therefore, for incidence data from 1998 to 2003, we imputed MenA case estimates by multiplying total reported meningitis cases (including all organisms) by the proportion of tested cases that were MenA positive based on the 2004 to 2010 data. We allowed the proportions of MenA positive cases to vary by epidemic and non-epidemic years because we observed a considerably higher proportion of MenA in epidemic years as compared with non-epidemic years in the 2004 to 2010 incidence data. We applied the age distribution of cases aged 1 to 29 years obtained from a study of MenA in Niger to estimate incidence by age [6]. For carriage estimates we used data from the largest and most recent carriage study from Burkina Faso. This large multiple cross-sectional carriage study of those 1 to 29 years of age was conducted by Kristiansen et al. in 2009 [9]. In order to maximize representativeness of our model to the meningitis belt, we only included data from the rural districts of the carriage study (n = 13,513 nasopharyngeal swabs), stratified by age and season for our model.

We used the following process to fit the model parameters to these data. First, to find a preliminary pair of WAIFW matrices, we explored numerous combinations of values that varied the degree of assortative mixing and the relative importance of each age group in transmitting MenA to other age groups. For each potential pair of dry and rainy season matrices we ran the model to equilibrium and determined which pair of matrices gave the best fit between observed and predicted age-specific prevalence of colonization during rainy seasons and dry seasons with minor epidemics prior to the introduction of MenAfriVac™. We then took the best-fit pair of matrices and used an iterative numeric process to refine the matrix parameters and get the best fit to the observed prevalence of colonization (Table S2). After fitting the WAIFW matrices, we then used the same iterative numeric process to refine the parameters for the rate of disease among the colonized, to get the best fit for the observed age-specific incidence in dry seasons with both minor (cumulative incidence <20 cases per 100,000) and with major (cumulative incidence >80 per 100,000) epidemics. To aid interpretation, we multiplied the rainy season WAIFW by the age-specific prevalence of carriage during a typical rainy season to obtain a matrix of the force of infection from each age group to each age group. Similarly, we created two force of infection matrices for the dry season: one for a dry season with a minor epidemic (when prevalence of carriage was low) and when for a dry season with a major epidemic (when prevalence of carriage was high).

### Implementation

We first divided the population into n = 361 age groups, by month of age from birth through 30 years of age, where those aged 30 years and older were treated as a homogenous age group. Within each age group, the set of partial differential equations that govern the model reduces to a set of ordinary differential equations. For computational simplicity we then divided the study time into discrete time steps (by calendar week) and implemented the model as a set of ordinary difference equations. Weekly time steps were chosen as being small enough to yield negligible variation between the difference and differential equations. During each week, we randomly selected a value for the stochastic paramater φ and then moved the population between the model states within each age group. We then incremented time by one week, aging the population by moving individuals from age n to age n+1 each month, with newborns entering the model at age n = 0.

We started the model on January 1^st^ 2010. We used 2010 census data to determine the age structure of Burkina Faso [26], and divided the population among the model states so that MenA transmission was in or nearly in equilibrium. Modeling and subsequent analyses were all implemented using SAS version 9.2 (SAS Institute Inc., Cary NC).

### Evaluating Model Fit

To evaluate how well the model fit observed data on the age-specific prevalence of carriage and age-specific incidence of disease, we ran 100 iterations of the model, for 40 years in each iteration. In each iteration we calculated the average simulated prevalence of carriage by age group during rainy seasons as well as during dry seasons that were not major epidemic years. We calculated the pseudo-R^2^ comparing observed carriage to simulated carriage for each run. We also calculated average age-specific annual incidence during seasons without major epidemics as well as during seasons with major epidemics. We then calculated the R^2^ comparing observed incidence to simulated incidence for each of those runs. Finally, we estimated the mean and standard deviation of the pseudo-R^2^ (for carriage) and the R^2^ (for incidence) across the 100 iterations.

### Scenarios for Vaccine Introduction

A primary objective for developing this model is to compare the relative impact of possible vaccination strategies of MenAfriVac™ in countries across the meningitis belt. We based our evaluation on nine different combinations of two of the most likely vaccination strategies: mass vaccination campaigns in select age groups; and integration of MenAfriVac™ into the current Expanded Program on Immunization (EPI) schedule, which in Burkina Faso consists of five contacts (birth, 2, 3, 4, and 9 months of age) [37]. The majority of the strategies we evaluated included a preliminary mass vaccination campaign of all 1 to 29 year olds, such as what was implemented in Burkina Faso in December, 2011. Specifically, we modeled primary mass campaign of all persons aged 1 to 29 years, plus either 1.) introduction of MenAfriVac™ into the EPI dose at nine months of age beginning at different time points (2, 5, 10, 25 years) following the initial mass-vaccination campaign, or 2.) additional periodic mass campaigns of select age groups. For comparison purposes, we also evaluated the impact of strategies consisting of one mass vaccination campaign of 1 to 29 year olds only, EPI at nine months only, and no vaccination. We assumed 89% vaccine coverage for mass campaigns and 80% coverage for EPI immunizations [9], [38], [39]. To compare the different scenarios for vaccine introduction, we calculated average overall annual incidence and incidence by age over 100 simulation runs for each vaccination strategy.

### Sensitivity Analyses

Because some parameters in our model are based on limited empirical data, we assessed whether our model conclusions are dependent on the specific parameter values we selected. We tested the sensitivity of the model to all the parameter values that were defined from the literature (rates of birth, death, recovery from colonization, recovery from disease, and waning of protection). As inference about the force of infection was one of the modeling objectives, we did not conduct sensitivity analyses on the force of infection parameters. We evaluated the model’s robustness to the specific values for individual parameters and for combinations of parameters.

We first ran 1,000 iterations of the model from 2010 through 2049, without vaccination, using the primary parameter values. From each simulation run we calculated the mean predicted annual incidence of invasive MenA by age group. For the sensitivity analyses we then ran 10,000 iterations of the simulation model. In each iteration, we randomly selected three parameters to vary; for these three parameters we randomly sampled values from a distribution defined by the mean and a standard error of 10%. We ran the model from 2010 through 2049 using the sampled values of the three parameters and the point estimates for all remaining model parameters. We calculated the mean predicted annual incidence of invasive MenA from each of the 10,000 runs, stratified by age group and epidemic size (minor vs. major). To assess whether the model was sensitive to any individual parameters, we tested whether the mean incidence varied significantly between all of the 10,000 runs where that parameter was varied and the 1,000 model runs using the primary parameter values, in each age group/epidemic size stratum, using the Šidák correction for multiple comparisons [40]. We repeated these comparisons using all two-way and all three-way combinations of parameters.

Our model assumes that vaccination with MenA vaccine is equivalent to natural infection in inducing immunity to future MenA colonization and disease. However, very high antibody responses to a single dose of MenA conjugate vaccine in pre-licensure clinical trials data suggest that MenAfriVac™ may induce a stronger immune response than natural infection [13]. We conducted sensitivity analyses to assess whether our conclusions about the relative benefits of different vaccination strategies would differ if MenAfriVac™ induces more immune protection than natural infection. For this, we added a ninth state to the model, representing vaccinated persons. In this model, vaccination moves persons to the vaccinated state, where they are completely immune to colonization. Vaccine protection wanes over time, moving subjects into the high protection, susceptible state. We repeated the comparisons of the impact of vaccination strategies on disease using this expanded model.

## Results

### Model Fit

**Figure 2:**
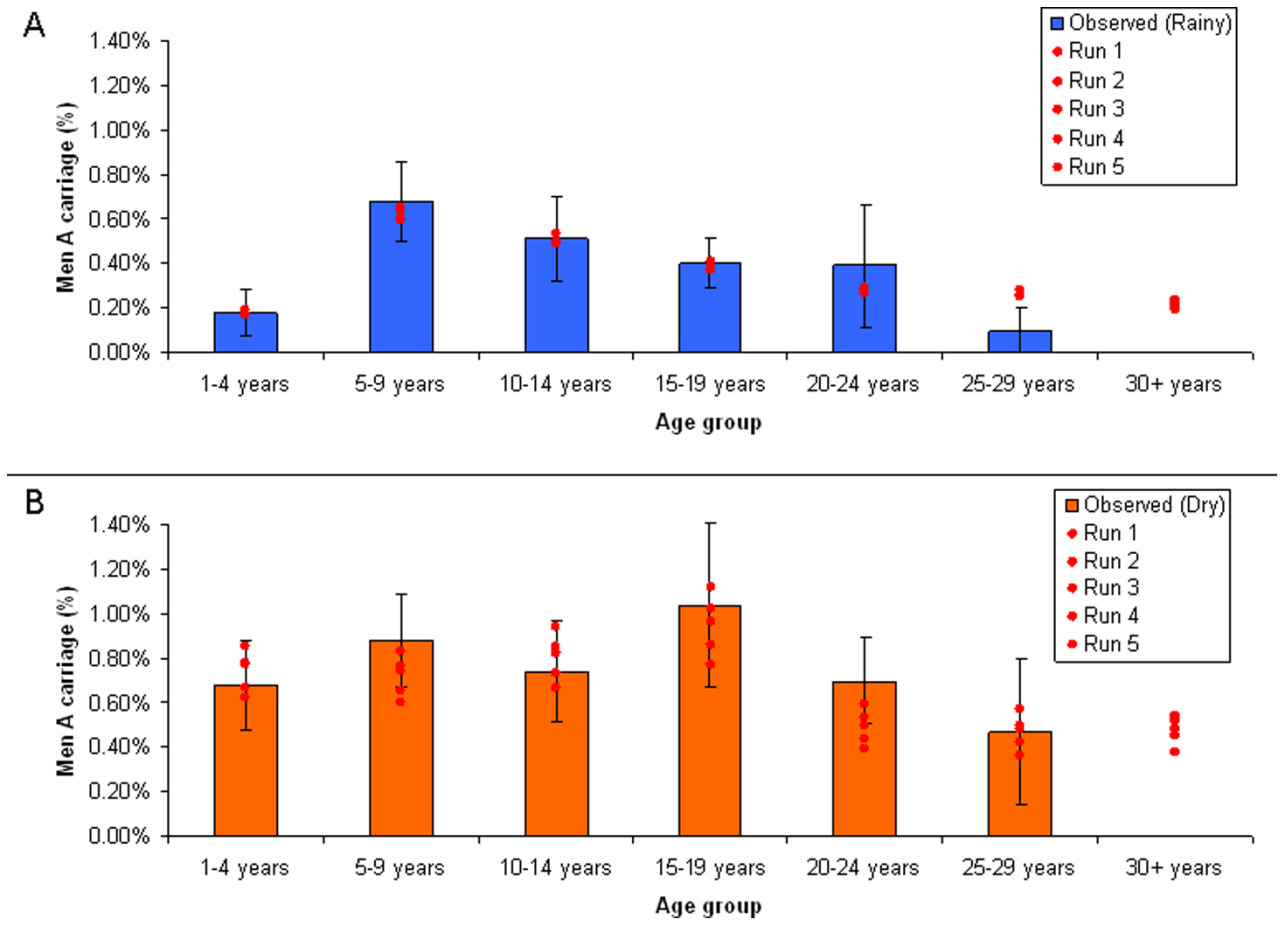
Prevalence of MenA Carriage in Rainy and Dry Seasons. a. Observed and simulated prevalence of MenA carriage in the rainy season prior to vaccine introduction, stratified by age group; vertical bars indicate standard errors. b. Observed and simulated prevalence of MenA carriage in the dry season prior to vaccine introduction, stratified by age group; vertical bars indicate standard errors.

**Figure 3:**
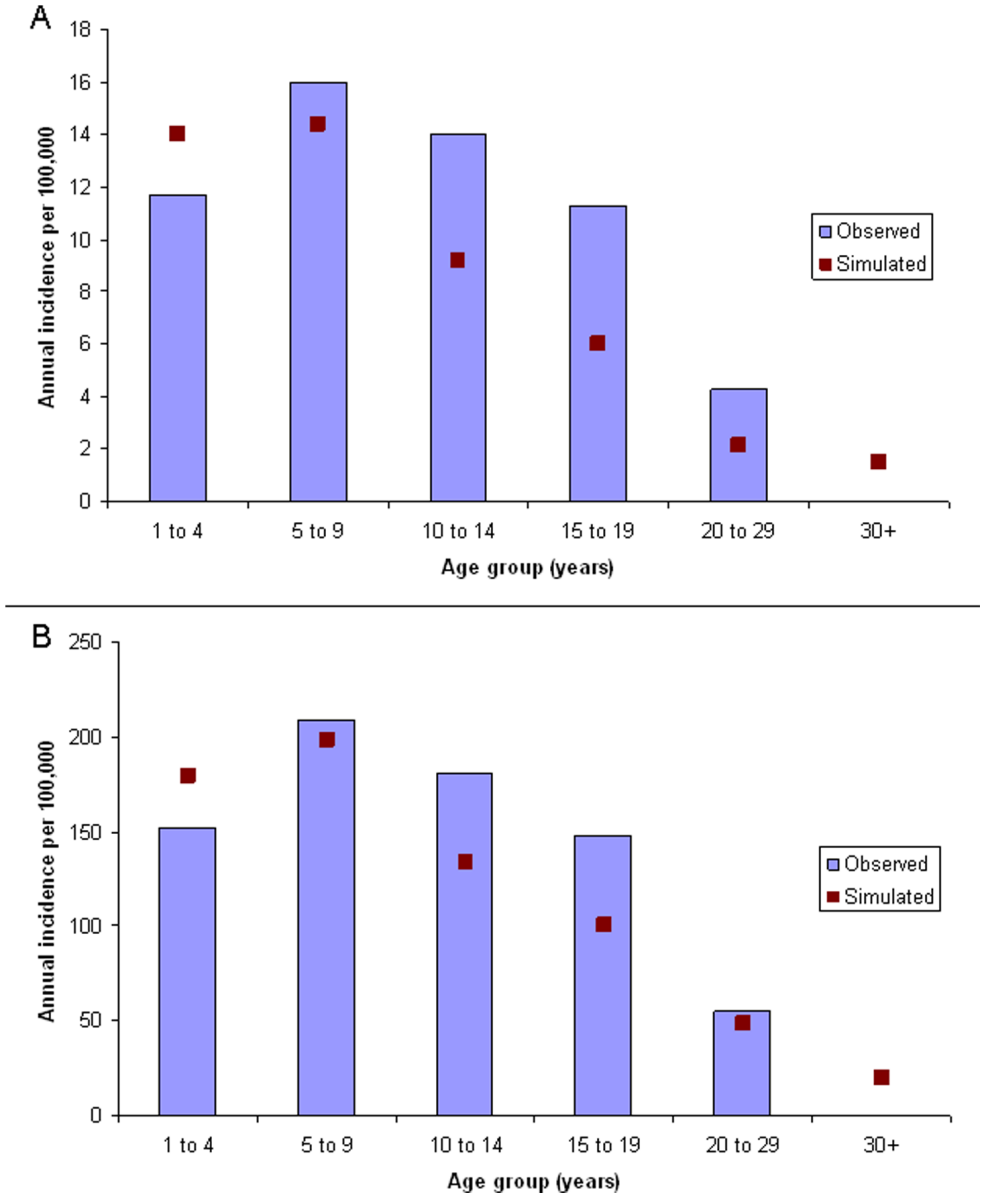
Incidence of MenA Meningitis in Minor and Major Epidemic Seasons. a. Observed and simulated incidence of MenA meningitis in minor epidemic seasons prior to vaccine introduction, stratified by age group. b. Observed and simulated incidence of MenA meningitis in major epidemic seasons prior to vaccine introduction, stratified by age group.

**Figure 4:**
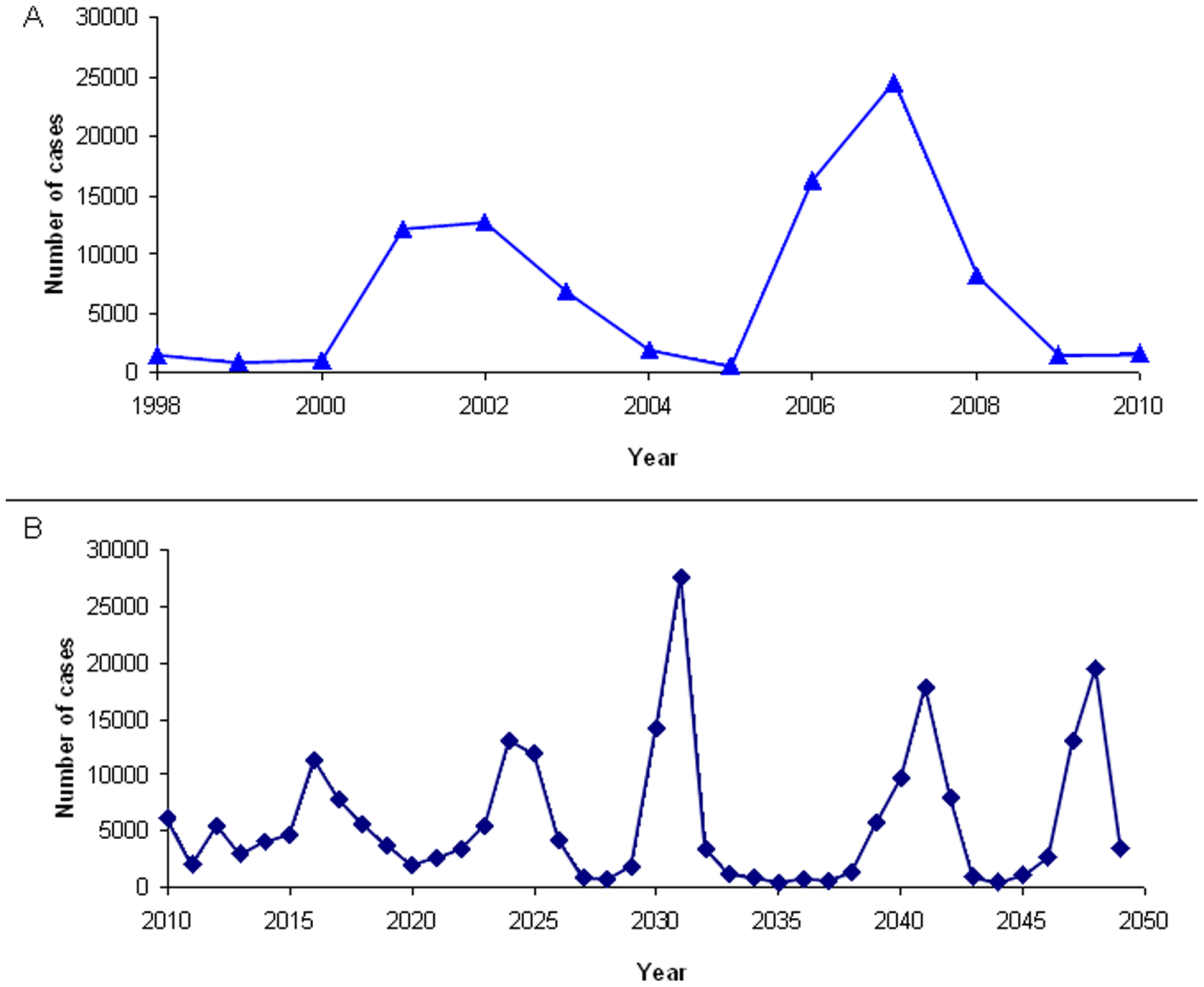
Estimated and Simulated MenA Case Counts, Burkina Faso. a. Estimated annual MenA case counts in Burkina Faso, 1998–2010. b. Simulated annual MenA case counts from a single simulation iteration, normalized to 2010 population.

The model accurately reproduces the unique epidemiology of meningitis in the meningitis belt, including smaller annual dry season epidemics as well as major multi-year epidemics every eight to twelve years. Specifically, simulated estimates of carriage prevalence (mean pseudo-R^2^ 0.84, SD = 0.054) (Figures 2a & 2b) and incidence rates (mean R^2^ 0.89, SD = 0.019) (Figures 3a & 3b) by age prior to introduction of MenAfriVac™ in Burkina Faso fit well to observed estimates. Incidence estimates from the model include large epidemics every eight to twelve years, during which incidence is ten or more times higher than during inter-epidemic years. Case counts simulated by the model in epidemic years range from approximately 15,000 to 25,000 cases, and case estimates from observed data range from approximately 13,000 to 25,000 (Figures 4a & 4b). Between large epidemic years and during the rainy season simulated and observed incidence and carriage estimates are low.

### Force of Infection

The best-fit WAIFW matrices suggest that meningitis transmission patterns between age groups prior to vaccine introduction differ between dry and rainy seasons. In the dry season, the force of infection on all age groups occurs predominantly through an assortative mixing pattern, where persons are primarily colonized through contact with members of the same age group or age groups which are similar in age (Table S2). For example, the annual combined force of infection on persons less than 5 years of age was 248.0 infections/100,000 persons, where 126.8 (51.1%) infections/100,000 persons were from persons less than 5 years of age. Alternatively, force of infection in the rainy season suggests that transmission is due to 5 to 12 year olds more than any other age group. Prevalence of carriage was similar between age groups, but differed considerably by season. Carriage prevalence was highest during major epidemics, when it was approximately eight times higher than prevalence during minor epidemics and approximately 19 times higher than predicted for rainy seasons.

### Predicted Impact of Vaccination

**Figure 5:**
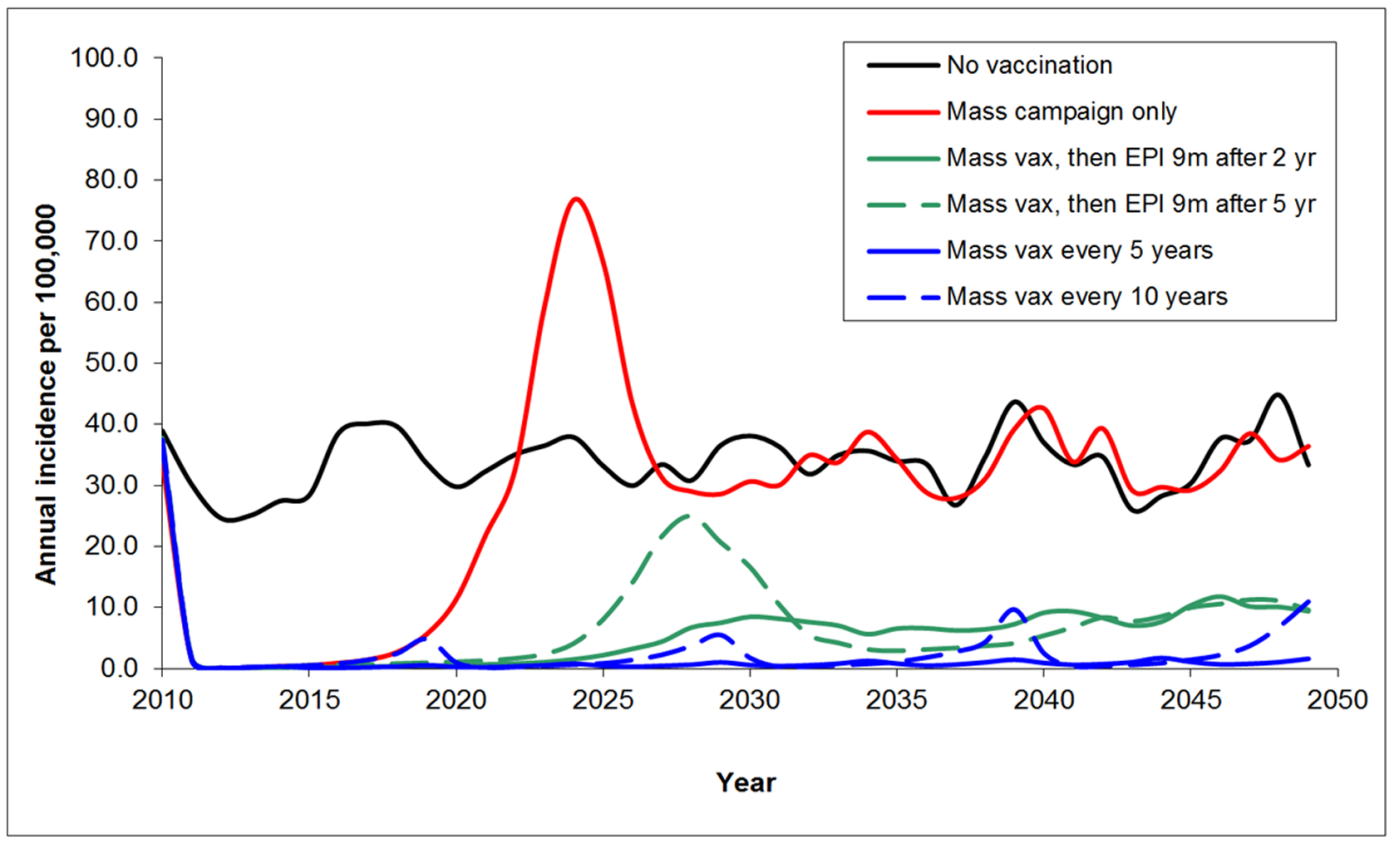
Annual incidence of invasive Neisseria meningitidis A under different vaccination scenarios, averaged across 100 simulation runs.

When averaged across 100 iterations of the simulation, the model predicts an average annual incidence of 34 cases per 100,000 population per year (Table 2, Figure 5). Due to the stochastic nature of the simulation, the timing of major epidemics varies from iteration to iteration; averaging across the iterations produces the “wavy” pattern seen in Figure 5 rather than periodic large epidemics that would be seen in a single iteration. Compared to the no-vaccination scenario, a primary mass vaccination campaign of all 1 to 29 year olds with no follow up strategy rapidly decreases disease incidence and maintains an incidence equilibrium <1 case per 100,000 for approximately ten years. After ten years disease incidence rises rapidly, reaching ∼80 cases per 100,000 per year. Following the peak, incidence declines rapidly to an average annual incidence of 34 cases per 100,000 per year, equal to the predicted equilibrium rate in the absence of vaccination.

**Table 2:** Estimated incidence of serogroup A meningitis under proposed vaccination strategies.

				Preliminary Mass Vaccination Campaign Plus Integration into EPI for All 9 Month Olds Starting After the Mass Vaccination Campaign at:				Preliminary Mass Vaccination Campaign Plus Additional Mass Vaccination Campaigns in Selected Age Groups	
Annual incidence per 100,000	No vaccination	EPI Only	Mass Campaign Only	2 years	5 years	10 years	15 years	1–5 year olds every 5 years	1–10 year olds every 10 years
**<1 yr**	68.6	15.0	55.0	9.5	11.8	15.7	23.2	4.1	6.5
**1–4 yr**	61.2	6.8	48.9	4.2	5.0	7.4	17.0	2.8	5.5
**5–9 yr**	64.7	11.6	54.0	6.0	7.4	15.3	24.0	2.0	5.3
**10–14 yr**	42.4	13.6	37.7	6.9	10.3	18.3	21.9	1.5	1.7
**15–19 yr**	27.6	14.6	20.8	8.7	13.5	13.5	13.0	1.2	1.6
**20–24 yr**	11.5	9.0	8.6	6.2	6.4	6.3	7.0	0.9	1.4
**25–29 yr**	8.9	8.0	6.6	5.7	5.7	5.6	5.6	0.9	1.4
**30+ yr**	6.8	5.9	6.2	4.6	4.8	4.6	4.6	1.0	1.9
**All ages**	33.9	9.4	28.1	5.8	7.3	10.0	13.6	1.6	3.0

All of the vaccination strategies that follow the primary mass campaign resulted in incidence equilibrium at or below 10 cases per 100,000 per year by the end of the 40 year period (Figure 5). However, there were differences between the strategies in the magnitude of incidence rates prior to reaching equilibrium. The strategies that most rapidly reach and maintain the lowest incidence equilibrium include additional mass vaccination campaigns of children 1 to 5 years old every five years, and mass campaigns of 1 to 10 year-old children every 10 years following the primary mass campaign. When mass vaccination of 1 to 5 year-olds is initiated five years following the primary campaign, an equilibrium incidence of ∼1 case per 100,000 is maintained over the entire 40-year period. When mass vaccination of 1 to 10 year olds is initiated 10 years following the primary campaign, equilibrium incidence reaches approximately one case per 100,000 per year, however, additional small peaks in incidence of approximately 10 cases per 100,000 per year occur every ten years with this approach.

Introducing vaccine into the EPI schedule at the nine month old visit following the primary mass campaign is also effective, although equilibrium incidence remains higher than what is predicted with periodic mass campaigns. Earlier introduction of vaccine into EPI is more effective at maintaining low equilibrium rates. When an EPI dose is initiated two years following the primary mass campaign, incidence equilibrium is maintained at approximately six cases per 100,000 across 40 years and across all age groups (Table 2). When introduction into EPI for all nine month olds begins five years following the primary mass campaign, a multi-year peak in incidence reaching 25 cases per 100,000 is predicted approximately 18 years following the primary campaign. Following this peak, incidence declines to an equilibrium rate of approximately 10 cases per 100,000 per year (Figure 5).

### Sensitivity Analyses

In sensitivity analyses of individual parameters, varying the rate of recovery from carriage yielded statistically significantly different mean incidence rates compared to the primary parameter values in four of the 10 age group/epidemic size combinations. Similarly, varying the immunity of high protection against disease yielded statistically significantly different mean incidence rates in one of the 10 age group/epidemic combinations. For analysis of pairs of parameters, varying rate of recovery from carriage with three other parameters (force of infection from outside populations, death rate, and immunity of high protection against carriage) yielded statistically significantly different rates in one or two age/epidemic combinations. No three-way combinations of parameters had significantly different mean incidence in any age/epidemic combinations. Importantly, none of the statistically significant differences were of practical significance, all being less than one case per 100,000 population per year.

As expected, the absolute number of cases prevented differed somewhat between the primary analyses and sensitivity analyses where MenAfriVac™ is assumed to be more protective than natural infection against future MenA colonization. However, the relative impact of different vaccination strategies was the same between the two models.

## Discussion

With the development and licensure of MenAfriVac™, Ministries of Health in the African meningitis belt need data that can predict optimum vaccination strategies to reduce MenA morbidity and mortality. We have developed an age-structured mathematical model that accurately reproduces the complex epidemiology of MenA in Burkina Faso, including annual minor epidemics and major multi-year epidemics every eight to twelve years. This is the second published model of MenA in Africa [41], and the first to incorporate age structure and varying levels of protection. With these advances, our model can predict the impact of several different vaccination strategies, and also provides general insights into the epidemiology of MenA in the African meningitis belt.

First, prior hypothetical models of MenA have suggested that seasonal differences in the incidence of invasive MenA may be due to seasonal changes in the rate at which susceptible persons become colonized (i.e., the force of infection), in the probability of invasive disease among colonized persons, or both [42], [43]. In our model we allowed both force of infection and the probability of disease among colonized persons to vary seasonally. We found that the observed changes in incidence could be explained by seasonal variation in the force of infection, as opposed to seasonal changes in the probability of disease among the colonized. Our model thus suggests that dry season MenA epidemics are driven by changes in contact patterns or in susceptibility to colonization given exposure (the two components of the Who Acquires Infection from Whom matrix). These findings are consistent with the model of Irving et al. [41], which found that seasonal changes in transmissibility of meningococci are more important than seasonal changes in the rate of progression to disease.

Second, some studies of MenA epidemics have postulated that the eight to twelve year cycle of major MenA epidemics is caused by the periodic emergence of hyper-virulent strains of MenA [44], [45]. Our model assumed no changes in the pathogen, and was able to reproduce these eight to twelve year epidemic cycles simply as a function of changes in population susceptibility (due to newly susceptible persons born into the population and to waning immunity among formerly colonized individuals). Thus, the dynamics of MenA in the African meningitis belt may be primarily driven by changes in population susceptibility over time rather than by changes in the pathogen.

Third, the force of infection estimates from our model indicate that the patterns of transmission between age groups differ between epidemic and non-epidemic seasons. Estimates are driven by both changes in rate of contact between age groups as well as changes in the probability of transmission given contact. Changes in rate of contact can be due to seasonal changes in crowding behaviors, seasonal migratory work patterns, and other factors. We found that in epidemic seasons, transmission occurs predominantly through an assortative mixing pattern, while in non-epidemic seasons transmission is primarily through 5 to 12 year olds.

When we applied our model to exploring the relative effectiveness of different possible MenA vaccination strategies, we found that both approaches we investigated – follow-up mass campaigns and integration into the EPI program – would reduce the incidence of invasive MenA compared to no vaccination. Overall, our model suggests that follow-up mass vaccination campaigns would reduce the population-level incidence of MenA more than would incorporating MenA into the routine EPI schedule. Mass vaccination campaigns of 1 to 5 year olds every five years prevented the most cases of all strategies we investigated. However, while integrating a single dose at nine months into EPI was less effective compared with mass campaigns, this strategy also offered considerable population-wide protection. The decision on the most appropriate strategy for any country or region will involve prioritization of a number of factors including cost and feasibility. Additionally, it will be important to evaluate coverage levels of the EPI program in the region as well. In countries where EPI coverage is low (<60% DTP3), immunization of new birth cohorts through the EPI integrated dose may not be sufficient to sustain protection in the population. Follow up mass campaigns are likely to be more successful in circumstances of low EPI coverage. Not surprisingly, we found that adding MenA vaccine to the EPI schedule would be most effective if done soon after an initial mass vaccination campaign. For every five years additional time post-mass vaccination that the EPI program is initiated, an additional three cases per 100,000 per year are estimated to occur.

The findings from this study were generated with data from Burkina Faso, and may not be generalizable to other countries in the meningitis belt. Additional limitations to the model include uncertainties inherent in the estimation and application of parameter values from the literature and the assumptions inherent in the model structure. In order to minimize and measure potential error, we modeled a range of values where it was necessary to make assumptions or extrapolate data. For example, data that describes rate of waning of immune response to *N. meningitidis* serogroup A disease or vaccination were largely unavailable from the literature. We extrapolated estimates for these values by applying immunogenicity data from studies following *N. meningitidis* serogroup C vaccination. We conducted extensive sensitivity analyses to assess whether our modeled conclusions are dependent on the particular parameter combinations we chose for the model. These analyses show that our model is robust to changes in the model parameters, and to the possibility that MenAfriVac™ induces superior protective relative to natural infection. Future modeling efforts would benefit greatly from long-term field studies of duration of immunity against disease and carriage from vaccination and natural infection; of carriage by season and age group for estimating forces of infection; and rates of recovery from colonization in vaccinated and unvaccinated individuals.

In the next five years, countries across the meningitis belt will introduce MenAfriVac™ by mass vaccination campaigns supported by the GAVI Alliance. However, the countries themselves will be responsible for both selecting and supporting long-term immunization strategies. While the optimal long-term vaccination plan should be governed by the capacity and needs of each country, the results of this model emphasize the need to vaccinate cohorts born after the mass campaigns to maintain long-term population protection. This is the first mathematical model that can be used by countries introducing MenAfriVac™ to support decision making about long-term vaccination strategies.

## Supporting Information

Table S1 **Model structure.**
(DOCX)

Table S2 **Estimated annual pre-vaccination force of infection from** ***N. meningitidis*** **infectious persons to persons with susceptible, no-antibody status and estimated annual prevalence of** ***N. meningitidis*** **colonization, stratified by age group.**
(DOCX)
