# Correction: Identifying Optimal Vaccination Strategies for Serogroup A *Neisseria meningitidis* Conjugate Vaccine in the African Meningitis Belt

**DOI:** 10.1371/journal.pone.0190188

**Published:** 2017-12-19

**Authors:** Sara Tartof, Amanda Cohn, Félix Tarbangdo, Mamoudou H. Djingarey, Nancy Messonnier, Thomas A. Clark, Jean Ludovic Kambou, Ryan Novak, Fabien V. K. Diomandé, Isaïe Medah, Michael L. Jackson

An error has been identified in the model code that meaningfully affects the study results. The code to implement the periodic campaigns in the simulation model was discovered to be incorrect. Instead of simulating a campaign once every five years (or once every ten years), the code was simulating 12 monthly campaigns every 5 years or every 10 years. This error made the vaccine coverage higher during the periodic campaigns than it should have been, which falsely increased the benefit of periodic campaigns relative to routine vaccination of infants. The authors have corrected the coding error and re-run the analyses. The correct analyses indicate that that periodic campaigns are not always superior to routine immunization, which conflicts with one of the study’s conclusions.

The second and third paragraphs in the “Predicted Impact of Vaccination” section of the Results have been updated. The correct text is: All of the vaccination strategies that follow the primary mass campaign resulted in incidence equilibrium at or below 14 cases per 100,000 per year by the end of the 40 year period (Fig 5). However, there were differences between the strategies both in the mean incidence once equilibrium is reached, and in the incidence rates prior to reaching equilibrium. Periodic vaccination mass campaigns of children result in average annual incidence of 8 to 13 cases per 100,000 after the year 2025. Campaigns every 5 years among children aged 1 to 5 years had low equilibrium incidence (8 cases per 100,000 annually). In contrast, campaigns every 10 years among children aged 1 to 10 years had the highest equilibrium incidence of any tested strategy (13 cases per 100,000), and allowed for decennial epidemics preceding each mass campaign. Introducing vaccine into the EPI schedule at the nine month old visit following the primary mass campaign is also effective at maintaining low disease incidence (Fig 5). Introducing vaccine to the EPI schedule 2 years after the mass campaign resulted in low equilibrium incidence (8 cases per 100,000 after the year 2025). Longer delays with introducing vaccine were associated with higher incidence after 2025, primarily because the delay allows enough children to remain susceptible that epidemics occur between 2025 and 2030 (15–20 years after the mass campaign).

The fifth paragraph of the Discussion has also been updated. The correct text is: When we applied our model to exploring the relative effectiveness of different possible MenA vaccination strategies, we found that both approaches we investigated—follow-up mass campaigns and integration into the EPI program—would reduce the incidence of invasive MenA compared to no vaccination. The best strategy is predicted to be either mass vaccination campaigns of 1 to 5 year olds every five years, or introducing vaccine to the EPI schedule two years after the initial campaign. Longer delays in adding vaccine to the EPI schedule can allow large outbreaks of MenA meningitis before the benefits of EPI vaccination are fully realized. The decision on the most appropriate strategy for any country or region will involve prioritization of a number of factors including cost and feasibility. Additionally, it will be important to evaluate coverage levels of the EPI program in the region as well. In countries where EPI coverage is low (<60% DTP3), immunization of new birth cohorts through the EPI integrated dose may not be sufficient to sustain protection in the population. Follow up mass campaigns are likely to be more successful in circumstances of low EPI coverage. Not surprisingly, we found that adding MenA vaccine to the EPI schedule would be most effective if done soon after an initial mass vaccination campaign. For every five years additional time post-mass vaccination that the EPI program is initiated, an additional three cases per 100,000 per year are estimated to occur.

Fig 5 has been updated using the corrected simulation model for the periodic catch-up campaigns. Please see the corrected Fig 5 here.

**Fig 5:**
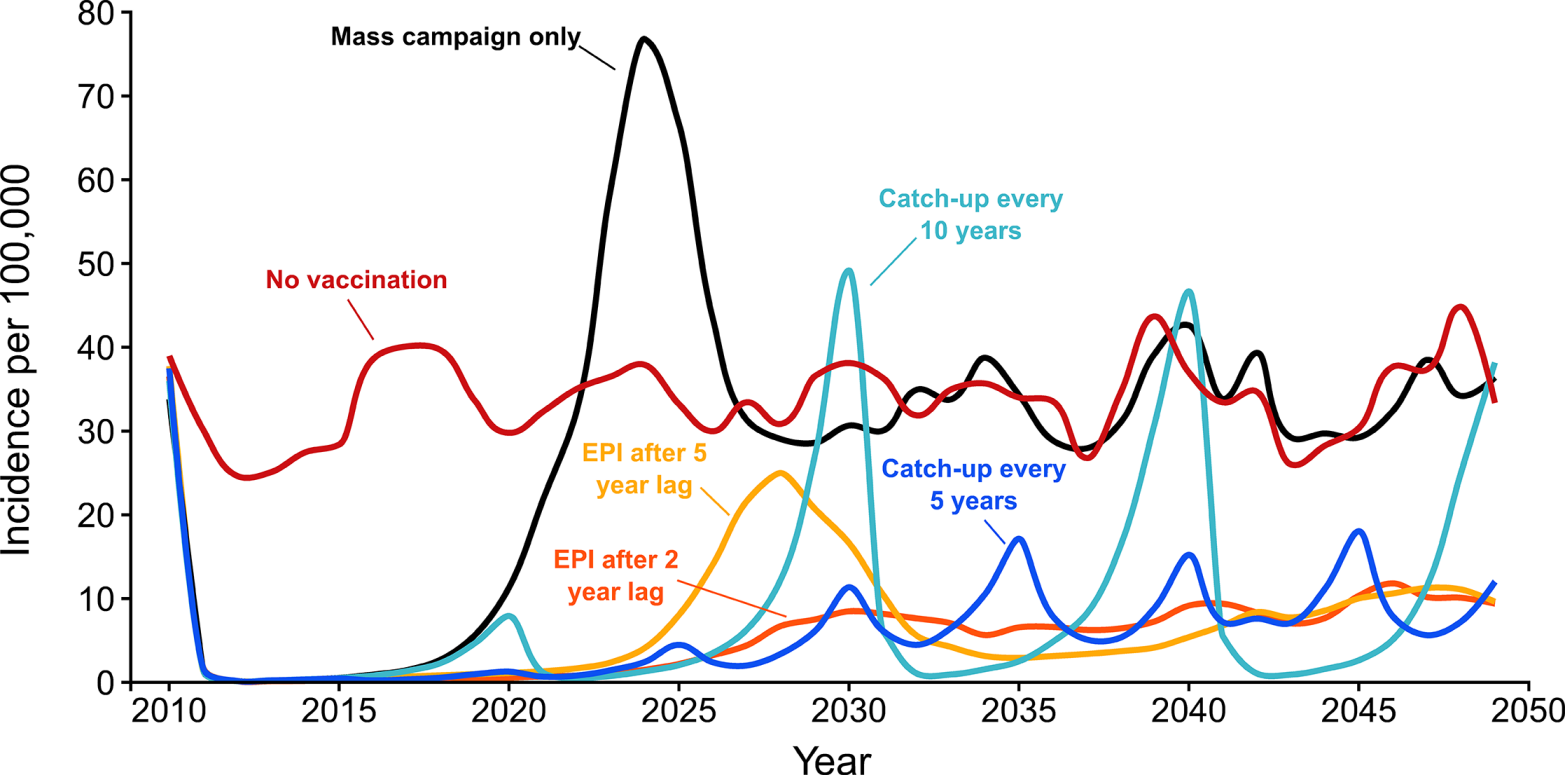
Annual incidence of invasive *Neisseria meningitidis* A under different vaccination scenarios, averaged across 100 simulation runs.

In Table 2, the “Preliminary Mass Vaccination Campaign Plus Additional Mass Vaccination Campaigns in Selected Age Groups” values in the columns “1–5 year olds every 5 years” and “1–10 year olds every 10 years” are incorrect. Table 2 has been updated using the corrected simulation model. Please see the corrected Table 2 here.

**Table 2:** Estimated incidence of serogroup A meningitis under proposed vaccination strategies.

				Preliminary Mass Vaccination Campaign Plus Integration into EPI for All 9 Month Olds Starting After the Mass Vaccination Campaign at:				Preliminary Mass Vaccination Campaign Plus Additional Mass Vaccination Campaigns in Selected Age Groups	
Annual incidence per 100,000	No vaccination	EPI Only	Mass Campaign Only	2 years	5 years	10 years	15 years	1–5 year olds every 5 years	1–10 year olds every 10 years
**<1 yr**	68.6	15.0	55.0	9.5	11.8	15.7	23.2	11.7	16.5
**1–4 yr**	61.2	6.8	48.9	4.2	5.0	7.4	17.0	8.9	15.0
**5–9 yr**	64.7	11.6	54.0	6.0	7.4	15.3	24.0	6.2	17.4
**10–14 yr**	42.4	13.6	37.7	6.9	10.3	18.3	21.9	6.5	5.8
**15–19 yr**	27.6	14.6	20.8	8.7	13.5	13.5	13.0	7.3	7.4
**20–24 yr**	11.5	9.0	8.6	6.2	6.4	6.3	7.0	5.4	5.2
**25–29 yr**	8.9	8.0	6.6	5.7	5.7	5.6	5.6	5.0	5.1
**30+ yr**	6.8	5.9	6.2	4.6	4.8	4.6	4.6	4.2	4.9
**All ages**	33.9	9.4	28.1	5.8	7.3	10.0	13.6	6.3	9.3

## References

[1] Tartof S, Cohn A, Tarbangdo F, Djingarey MH, Messonnier N, Clark TA, et al (2013) Identifying Optimal Vaccination Strategies for Serogroup A *Neisseria meningitidis* Conjugate Vaccine in the African Meningitis Belt. PLoS ONE 8(5): e63605 https://doi.org/10.1371/journal.pone.0063605 23671685 10.1371/journal.pone.0063605 PMC3650081

