# Supplementary material for: Identifying Optimal Vaccination Strategies for Serogroup A Neisseria meningitidis Conjugate Vaccine in the African Meningitis Belt
Source: PLoS One. 2013 May 9;8(5):e63605. doi: 10.1371/journal.pone.0063605 (PMC3650081; doi:10.1371/journal.pone.0063605)
Supplement: Table S1 — Model structure. (DOCX) [file pone.0063605.s001.docx]

**Supporting Information Table S1**

**Model structure**

The following set of partial differential equations defines the rates at which the simulated population moves between model states:

$$\frac{\partial NS}{\partial t}+\frac{\partial NS}{\partial a}=\mu(t,a)X(t)+\omega_{L}(a)LS(t,a)-\left[\nu(t)+\lambda(t,a)+\gamma(t,a)\right]NS(t,a)$$

$$\frac{\partial NC}{\partial t}+\frac{\partial NC}{\partial a}=\lambda(t,a)NS(t,a)-\left[\nu(t)+\rho_{C}+\sigma(a)+\gamma(t,a)\right]NC(t,a)$$

$$\frac{\partial LS}{\partial t}+\frac{\partial LS}{\partial a}=\rho_{c}NC(t,a)+\omega_{H}(a)HS(t,a)-\left[\nu(t)+\omega_{L}(a)+\lambda(t,a)(1-\alpha_{L})+\gamma(t,a)\right]LS(t,a)$$

$$\frac{\partial LC}{\partial t}+\frac{\partial LC}{\partial a}=\lambda(t,a)(1-\alpha_{L})LS(t,a)+\left[\nu(t)+\rho_{C}+\sigma(a)(1-\beta_{L})+\gamma(t,a)\right]LC(t,a)$$

$$\frac{\partial HS}{\partial t}+\frac{\partial HS}{\partial a}=\rho_{C}\left[LC(t,a)+HC(t,a)\right]+\rho_{D}D(t,a)+\gamma(t,a)[LS(t,a)+NS(t,a)]-\left[\nu(t)+\omega_{H}(a)+\lambda(t,a)(1-\alpha_{H})\right]HS(t,a)$$

$$\frac{\partial HC}{\partial t}+\frac{\partial HC}{\partial a}=\lambda(t,a)(1-\alpha_{H})HS(t,a)+\gamma(t,a)[LC(t,a)+NC(t,a)]-\left[\nu(t)+\rho_{C}+\sigma(a)(1-\beta_{H})\right]HC(t,a)$$

$$\frac{\partial D}{\partial t}+\frac{\partial D}{\partial a}=\sigma(a)\left[NC(t,a)+(1-\beta_{L})LC(t,a)+(1-\beta_{H})HC(t,a)\right]-\left[\nu(t)+\rho_{D}\right]D(t,a)$$

In which:

- NS, NC, LS, LC, HS, HC, and D, are population states, where N=No protection, L = Low protection, H = High protection, S = Susceptible, C = Colonized, and D = Diseased; X(t) is the total population.
- μ(t,a) and ν(t) are time-dependent birth and death rates, respectively. Birth rate also depends on age as individuals are only born into the age=0 group
- ω_L_(a) is the age-dependent rate at which low protection wanes to no protection and ω_H_(a) is the age-dependent rate at which high protection wanes to low protection
- λ(t,a) is the time- and age-dependent force of infection
- γ(t,a) is the time- and age-dependent rate of vaccination
- σ(a) is the age-dependent rate of invasive disease among colonized persons
- α_L_ and α_H_ are the efficacy of low and high antibody at preventing colonization
- β_L_ and β_H_ are the efficacy of low and high antibody at preventing invasive disease
- ρ_C_ and ρ_D_ are the rates of recovery from colonization and invasive disease, respectively
