# Supplementary material for: Identifying Optimal Vaccination Strategies for Serogroup A Neisseria meningitidis Conjugate Vaccine in the African Meningitis Belt
Source: PLoS One. 2013 May 9;8(5):e63605. doi: 10.1371/journal.pone.0063605 (PMC3650081; doi:10.1371/journal.pone.0063605)
Supplement: Table S2 — Estimated annual pre-vaccination force of infection from N. meningitidis infectious persons to persons with susceptible, no-antibody status and estimated annual prevalence of N. meningitidis colonization, stratified by age group. (DOCX) [file pone.0063605.s002.docx]

**Supporting Information Table S2**

**Estimated annual pre-vaccination force of infection from *N. meningitidis* infectious persons to persons with susceptible, no-antibody status and estimated annual prevalence of *N. meningitidis* colonization, stratified by age group.**

| Force of infection (instantaneous rate of infections per 100,000 among no antibody, susceptibles) by age: | | | | | |  |
| --- | --- | --- | --- | --- | --- | --- |
| **Rainy season** | | **Age of infectious (j)** | | | | **Total Rate** |
|  |  | **<5** | **5-12** | **13-19** | **20+** |  |
| **Age of susceptible (i)** | **<5** | 3.9 | 15.8 | 12.9 | 6.4 | 39.0 |
|  | **5-12** | 15.1 | 59.3 | 34.1 | 8.4 | 116.9 |
|  | **13-19** | 7.1 | 46.0 | 29.0 | 20.5 | 102.6 |
|  | **20+** | 8.5 | 37.6 | 19.2 | 17.9 | 83.2 |
|  | **Carriage prevalence:** | 0.20% | 0.49% | 0.30% | 0.18% |  |
|  |  |  |  |  |  |  |
| **Minor epidemic (Dry Season)** | | **Age of infectious (j)** | | | | **Total Rate** |
|  |  | **<5** | **5-12** | **13-19** | **20+** |  |
| **Age of susceptible (i)** | **<5** | 126.8 | 48.1 | 47.8 | 25.2 | 248.0 |
|  | **5-12** | 144.7 | 41.7 | 66.6 | 24.4 | 277.3 |
|  | **13-19** | 83.9 | 232.9 | 235.3 | 44.5 | 596.7 |
|  | **20+** | 49.6 | 89.5 | 76.0 | 138.1 | 353.2 |
|  | **Carriage prevalence:** | 0.72% | 0.65% | 0.94% | 0.41% |  |
|  |  |  |  |  |  |  |
| **Major epidemic (Dry Season)** | | **Age of infectious (j)** | | | | **Total Rate** |
|  |  | **<5** | **5-12** | **13-19** | **20+** |  |
| **Age of susceptible (i)** | **<5** | 1,003.7 | 391.4 | 372.9 | 206.6 | 1974.7 |
|  | **5-12** | 1,146.2 | 337.9 | 521.7 | 199.6 | 2205.4 |
|  | **13-19** | 661.9 | 1,921.2 | 1,860.7 | 370.4 | 4814.2 |
|  | **20+** | 388.4 | 733.7 | 596.1 | 1,165.1 | 2883.3 |
|  | **Carriage prevalence:** | 5.70% | 5.35% | 7.44% | 3.49% |  |
|  |  |  |  |  |  |  |
